# Feeding Intolerance in Children with Severe Impairment of the Central Nervous System: Strategies for Treatment and Prevention

**DOI:** 10.3390/children5010001

**Published:** 2017-12-22

**Authors:** Julie Hauer

**Affiliations:** 1 Boston Children’s Hospital, Division of General Pediatrics, Harvard School of Medicine, 300 Longwood Ave, Boston, MA 02115, USA; julie.hauer@childrens.harvard.edu; Tel.: +1-978-448-3388; 2 Seven Hills Pediatric Center, 22 Hillside, Groton, MA 01450, USA

**Keywords:** pediatric, neurological impairment, feeding intolerance, retching, visceral hyperalgesia, central neuropathic pain, autonomic dysfunction, disability, pediatric palliative care, symptom management

## Abstract

Children with severe impairment of the central nervous system (CNS) experience gastrointestinal (GI) symptoms at a high rate and severity, including retching, vomiting, GI tract pain, and feeding intolerance. Commonly recognized sources of symptoms include constipation and gastroesophageal reflux disease. There is growing awareness of sources due to the impaired nervous system, including visceral hyperalgesia due to sensitization of sensory neurons in the enteric nervous system and central neuropathic pain due to alterations in the thalamus. Challenging the management of these symptoms is the lack of tests to confirm alterations in the nervous system as a cause of symptom generation, requiring empirical trials directed at such sources. It is also common to have multiple reasons for the observed symptoms, further challenging management. Recurrent emesis and GI tract pain can often be improved, though in some not completely eliminated. In some, this can progress to intractable feeding intolerance. This comprehensive review provides an evidence-based approach to care, a framework for recurrent symptoms, and language strategies when symptoms remain intractable to available interventions. This summary is intended to balance optimal management with a sensitive palliative care approach to persistent GI symptoms in children with severe impairment of the CNS.

## 1. Introduction

Retching, vomiting, and gastrointestinal (GI) pain are frequent and significant problems experienced by children with severe impairment of the central nervous system (CNS), often referred to as children with severe neurological impairment (SNI) [1,2,3,4,5,6,7,8,9]. The GI tract is one of the most frequent sources of pain in children with SNI despite treatment of common sources such as gastroesophageal reflux disease (GERD) and constipation [2,3,4,5,6,10]. Pain attributed to the GI tract is noted to have a high pain intensity of 7.5 (from a 0–10 scale), second only to pain of unknown cause, with significantly higher rates of pain in children already receiving treatment for GERD or impaired GI tract motility [2,5,10]. Many continue to have recurrent symptoms despite evaluation and treatment directed at such problems, and such children may experience repeated testing in search of a cause.

These problems can result in feeding intolerance that for some can become a recurrent and persistent problem despite management of commonly recognized sources. This was identified as one of the most common problems in children with progressive genetic, metabolic, or neurologic conditions with no cure, with pain, sleep, and feeding difficulties identified by parents as the three most common problems, with symptoms often not well controlled [11].

This article will focus on GI symptoms that are recurrent and persistent in children with SNI. The goal of this article is to provide evidence-based suggestions to guide empirical treatment trials. This includes a review of how the altered CNS can contribute to such symptoms and interventions to modify how these symptoms are generated.

## 2. A Framework

Children with SNI and recurrent retching, emesis, and feeding intolerance often have multiple sources that contribute to these symptoms. As a starting framework, some are common management issues such as constipation and minimizing excessive calories, some are due to the altered CNS, and some are acute problems that can worsen the chronic symptoms. Further considerations include:

- Problems with tests that are “fixable”: urinary tract infection (UTI), acute pancreatitis, cholecystitis, nephrolithiasis, volvulus, helicobacter pylori.
- Problems with tests that can be modified and empirically managed: GERD, dysmotility.
- Problems without tests due to the altered nervous system that can be modified, require empirical trials, and can remain intractable: autonomic dysfunction, altered enteric nervous system, visceral hyperalgesia, central neuropathic pain, altered vomiting center in the medulla.
- Problems due to a wide range of needs in the same group of children: calorie and fluid estimates.

## 3. Testable Causes

Causes of acute emesis and abdominal pain typically have a diagnostic test to then guide treatment. Causes to consider include acute gastroenteritis, urinary tract infection, acute pancreatitis, nephrolithiasis, cholecystitis, volvulus, superior mesenteric artery syndrome, or adhesions. When symptoms persist, causes due to the altered nervous system (Table 1) are important to consider in a child with negative tests and insufficient benefit from treatment for such problems as GERD.

## 4. Causes Due to the Altered Nervous System

### 4.1. Visceral Hyperalgesia and Central Neuropathic Pain

Visceral hyperalgesia is an altered threshold to pain generation in response to a stimulus in the GI tract [12]. As a result, a normal stimulus, such as distention and pressure within the GI tract, can result in pain. Alternatively, tissue inflammation or injury, such as from GERD or surgery to the GI tract, may result in sensitization of visceral afferent pathways, with resulting visceral hyperalgesia.

Central neuropathic pain can develop when injury or disease of the CNS involves the thalamus or spinothalamic tract [13,14,15]. Symptoms due to this cause of pain include visceral pain associated with distention of the GI tract and bladder, described by one adult as feeling “like my bowels will explode” [16].

Both are reviewed together given the inability to know when GI tract pain is due to sensitization of sensory neurons in the enteric nervous system or a result of altered descending inhibitory control. Both can be suggested by (1) pain, retching and vomiting associated with gastrostomy tube (G-tube) feedings as a result of decreased gastric volume threshold to symptom generation; (2) pain associated with intestinal gas and jejunostomy tube (J-tube) feedings, suggesting pain with intestinal distention; (3) pain associated with flatus and bowel movements suggesting pain associated with colonic distention; and (4) persistent symptoms despite treatment of an identified problem such as GERD. The inability to tolerate a reasonable feeding rate may indirectly indicate a decreased threshold to symptom generation from GI tract distention.

Two considerations to management are: interventions that lessen GI tract distention and medications that lessen symptom generation. The former includes alterations in feeding volume rate, a review of calorie estimates, and G-tube venting, as examples. The later can include medication trials directed at visceral hyperalgesia and central neuropathic pain (Table 1).

Medication options for both include gabapentin and tricyclic antidepressants (TCAs) [13,14,15,16,17,18,19]. In addition, use of gabapentin for persistent pain in children with SNI resulted in a significant reduction of associated GI symptoms, including decreased emesis and retching, improved feeding tolerance, weight gain, and change from J-tube to G-tube feedings [17,20,21]. Clonidine also has suggested benefit in reducing pain perception during gastric and colonic distension [22].

### 4.2. Autonomic Dysfunction

Autonomic dysfunction, also called dysautonomia, paroxysmal sympathetic hyperactivity, autonomic storming, or sympathetic storming, can be due to alterations in the hypothalamus in children with SNI. Symptoms include tachycardia, hyperthermia, flushing of skin, abdominal pain, vomiting, bowel dysmotility, constipation, urinary retention, excessive sweating, increased salivation, posturing, and agitation [23,24,25].

Literature is limited to case reports, predominantly in patients with hypoxic and traumatic brain injury, with mixed results for interventions reported including benzodiazepines, bromocriptine, clonidine, oral and intrathecal baclofen (ITB), beta antagonists, and morphine sulfate [26,27,28,29]. More recently reported interventions include gabapentin and pregabalin [25,30]. In addition to scheduled gabapentinoid or clonidine, children with intermittent “autonomic storms” may benefit from as needed clonidine, benzodiazepine, or morphine sulfate during these episodes.

### 4.3. Emetic Reflex and Vomiting Center

The emetic reflex is the mechanism by which the CNS protects the body from potentially toxic substances. This complex reflex involves input to the vomiting center (VC), the final pathway. Receptors in the VC include histamine (H-1), acetylcholine (Ach), and serotonin (5HT-2) [31]. Alterations in the GI tract can stimulate the VC, predominantly mediated through the vagus and sympathetic nerves. The receptors involved include various serotonin (5HT) receptors in the GI tract including 5HT-4 prokinetic receptors and dopamine (D-2) receptors in the gastric wall. Substance P has also been identified in the GI tract, a neurotransmitter that induces vomiting through stimulation of the neurokinin (NK-1) receptors located in the chemoreceptor trigger zone.

Stimulation of these receptors can involve either distention or inflammation in the GI tract. Distention is detected by mechanoreceptors and inflammation by chemoreceptors. The goal is to treat sources when possible and block triggered receptors when the source cannot be fully eliminated. As an example, altered motility can result in recurrent distention of the intestines and colon. Treatment to modify motility is intended to minimize development of symptoms, with medications that target involved receptors then lessening symptom generation.

Medications that block these receptors include cyproheptadine and ondansetron (Table 2). Some medications, such as cyproheptadine, block more than one receptor involved with triggering the emetic reflex. Cyproheptadine has been identified to improve feeding tolerance, decrease emesis, and decrease retching including after fundoplication [32,33].

## 5. Over-Feeding

Over-feeding was identified as the third most common contributor to feeding intolerance, behind formula osmolarity and feeding rate [34]. Children with SNI have significantly lower energy expenditure as assessed by indirect calorimetry [35,36]. Calorie estimates using guidelines for children with cerebral palsy (CP) can over-estimate energy requirements in children with SNI by 30–40% [36,37,38,39]. Factors that contribute to this over-estimation include that children with SNI often have decreased muscle mass, which can account for 20–30% of resting energy expenditure [35]. Other factors that also decrease energy expenditure by approximately 25% include limited movement of extremities and hypothermia [36,40].

Guidelines for energy requirements have been established for children with CP. Using length, energy requirements are typically 12–15 kcal/cm for those who are ambulatory and 10–11 kcal/cm for those who are non-ambulatory, with some needing only 6–9 kcal/cm or less as a result of those factors that further lower energy expenditure [38,39]. Energy expenditure can also be determined by estimating the physical activity coefficient factor to use with the resting energy expenditure (REE, in kcal/day). Many children with SNI require a factor of 0.8 (kcal/day = REE × 0.8), with some as low as 0.5–0.6 [38]. This is in contrast to a factor of 1.5 to 1.6 for a typically developing, healthy child and a factor of 1.1 for a child who is non-ambulatory due to CP.

The goal in children with feeding tubes who remain life-long dependent on others to estimate calorie intake is to avoid excessive weight gain and to minimize associated symptoms of over-feeding, including retching, emesis, and GI tract pain. Children at highest risk for over-estimating intake include children with:

- Limited movement of extremities at baseline.
- A decrease in movement following improvement in symptoms (increase in baseline tone and movement are common features associated with pain) [20].
- Hypothermia due to impaired central regulation of body temperature.
- Gradual decline in activity when there is a decline in function and health over months to years.

Fluid needs can also be overestimated in children with a low metabolic rate, given that energy expenditure accounts for a portion of fluid needs. Of note, fluid requirement calculations based on weight were developed in ambulatory individuals with higher metabolic rates. This recognition allows a feed reduction trial without a need to maintain the same total fluid volume, when the goal of the trial is to determine if this will reduce emesis and GI tract pain.

A discussion regarding a reduction in calories is best approached gently, given the symbolic nature of feeding and nutrition. Language that may benefit families is an acknowledgment that it can seem counter intuitive to suggest a reduction in feeds as potentially beneficial for a child. Taking time to reflect on information can lessen associated fear and allow concerns to be adequately explored.

Some children will benefit from a feeding reduction trial when emesis, retching, or pain localized to the GI tract persists, following a comprehensive assessment for testable/treatable sources and management of common problems such as GERD and constipation. A 30% reduction of feeds is suggested to ensure an adequate trial while monitoring for benefit. A dietician can determine the need for micronutrient and protein supplementation when feeds are decreased. Along with monitoring weight, parents can monitor for changes in how clothing is fitting. This author has observed children with minimal to no change in weight when symptoms are improved following a reduction in feeds. This may reflect improved retention of intake due to decreased emesis and decreased metabolic expenditure due to a decrease in tone and movement when pain is reduced [20,38].

## 6. Empirical Trials

Many of the interventions listed in Table 2 require an empirical trial, including medications directed at causes due to the altered nervous system given the lack of diagnostic tests. Information regarding empirical trials directed at these problems, including dosing guidelines, is reviewed in detail in this clinical report from the American Academy of Pediatrics [16].

### 6.1. Medication Trials

Recurrent emesis and GI tract pain can often be improved, though in some not completely eliminated. The optimal plan can involve significant time and effort in those with recurrent symptoms and is best guided by broader considerations [16]. Information to consider when starting an empirical medication trial includes (1) response to previous medications; (2) interaction with other drugs; (3) initial dose; (4) the need for titration to minimize adverse effects; (5) the minimal initial dose; and (6) adverse effects [16]. Monitoring will determine whether there is adequate benefit and, if not, if a second medication will be added while continuing the first medication. As an example, the use of two or more medications with different mechanisms of action may reduce symptoms generated by neuropathic pain and reduce adverse effects if synergistic benefit allows for dose reduction [16].

### 6.2. Home Care Plans

Medications can modify symptoms generated by the altered nervous system, though breakthrough symptoms can occur due to the inability to eliminate the cause. Parents can be empowered with care plans to utilize for breakthrough symptoms. Interventions can be tried and then the care plan modified as information is gained. As an example, some children will benefit from use of an as needed suppository when retching or GI tract pain recurs, with such a trial determining if this is helpful for a specific child. Such an intervention can lessen colonic distention if the intervention results in a bowel movement, given the inability to know if there was incomplete evacuation with the last bowel movement.

Information to consider and document in a care plan includes:

- Presenting symptoms (vomiting, retching, pain).
- Initial routine interventions (vent G-tube).
- Interventions for triggers due to GI tract distention (use as-needed suppository, use enema if no results within 1-h of suppository, hold feeds for 2 h, hold feeds and give electrolyte replacement overnight, reduce total feeds/fluids).
- Use of as-needed medications (options include as needed antacid, ondansetron, clonidine, or benzodiazepine).
- When to call (call the clinic during the day or the on-call clinician after hours if symptoms persist despite use of the interventions outlined).

As an example, this was a beneficial strategy for an 18-year-old with SNI, thought to be due to birth anoxia, with recurrent episodes of abdominal distention and pain. Through trial and error, the following care plan was developed. This allowed care during events to remain at home and lessened repeat testing.

Initiate care plan for the following symptoms: persistent abdominal distention with discomfort or vomiting.

- Vent G-tube, hold use × 2 h.
- If no stool that day, give fleet enema.
- Give pedialyte at 40 mL/h × 4 h, then 70 mL/h × 24–36 h.
- Give acetaminophen every 4–6 h × 3–4 doses.
- Use morphine as needed, as often as every 4 h.
- Update team.

## 7. Acute on Chronic Symptom Events

If the frequency or severity of events increases, there is a balance between two considerations: assessment for a new acute source while considering a modification to a medication dose or an additional empirical medication trial directed at the sources due to the altered nervous system. Past experience, along with parental preference, will guide this balance. In a child with repeated negative tests, there may be a shared decision to focus on modifying the chronic care plan, given the decreased likelihood of the same tests identifying a new problem. A supportive and flexible approach can guide decisions in the child with recurrent symptoms. Language at such times might include the following:

- “Most recent tests have been negative, and I have the option of increasing the dose of his gabapentin or clonidine (I have the option to add a medication that targets the same symptoms in a different way). It might make sense to adjust medications while considering tests. You know your son best and I want you to feel comfortable with the plan. What makes best sense to you at this time?”
- “I imagine this is hard as I talk about sources that can be improved but not fixed”.
- “It must be hard as I talk about his nervous system being a reason for these symptoms when I don’t have a test to tell you with certainty. What are your thoughts as we discuss this information?”

## 8. Intractable Feeding Intolerance and Features at End of Life

Persistent feeding intolerance following various interventions is an intensely stressful experience for families of children with SNI. The assessment and management often reflect months of repeated studies and various interventions. Though intractable feeding intolerance is not common, the inherent challenges deserve consideration.

It can be beneficial, as further interventions outlined in Table 2 are considered, to simultaneously be mindful that this may be part of an irreversible decline. Language at such times can include, “I hope for as much benefit from this next trial, although I also want you to be prepared that we might not have the hoped-for benefit. What is important to you as we consider these possibilities?” [41].

This can be challenging, as children with SNI often experience decline over a long period, increasing the likelihood of testing and intervention trials of an irreversible problem. Alterations in GI tract function that is under the control of the CNS [42,43] may be a result of ongoing neuronal apoptosis. Changes in the CNS may account for why some children with SNI develop feeding intolerance that is not amenable to medical interventions [44]. Other features that can suggest changes in CNS function include altered alertness (pathways involved with arousal), altered regulation of body temperature and heart rate (autonomic function), frequency of seizures, level of comfort (thalamus), and altered vasomotor tone resulting in peripheral edema (hypothalamus and medulla), along with the regulation of GI tract function (hypothalamus and medulla). The development of persistent peripheral edema is the most likely to indicate irreversible changes, reported as a feature in the last weeks to months of life in children with SNI, and as a terminal feature in adults with multiple sclerosis and CNS tumors [45,46].

These considerations are important for parents to minimize over-testing at a time of diminishing benefit. Palliative care teams can provide support and guidance throughout this process. Suggested language includes: “These features can be due to changes in the brain. This means that the problems we are seeing might not improve with available treatment. As we try the next intervention we discussed, what is most important to you at this time?”, “I know that comfort is an important goal. I worry that it has been difficult to meet this goal or that it will only be possible with increased sedation or a decrease in total feeds. What are your thoughts?” [41]. Discussions may result in a shared decision to redirect goals and change care plans, such as reconsidering the role of further testing, resuscitation, and hospitalization.

The use of medical fluids and nutrition can be reviewed when there is persistent symptom burden due to tube feedings that is not alleviated with available interventions. At such times the use of a feeding tube can be viewed as a life-sustaining technology that may be prolonging suffering. This allows one to consider the amount of benefit and harm when technology is becoming more burdensome, even when that technology is “routine” to use. This is also a time to celebrate the years of benefit that were provided by the feeding tube as development of harm from this intervention is considered.

The process of considering peripheral nutrition as an alternative means of providing medical nutrition and hydration in children with SNI can be considered in the context of how a child is doing overall. Peripheral nutrition has greater likelihood of harm in the child with a variety of features that might be due to alterations in the CNS. A family may view such technology as burdensome and as prolonging suffering, when a child has experienced a decline in other areas of health and function. It is also helpful to consider what goal is intended by the intervention. Examples of goals are to improve comfort, to restore health, or to maintain life.

Discontinuing medical nutrition and hydration remains challenging and controversial, because of the symbolic significance of nutrition, the myths about dehydration and “starvation,” and the under-recognition of symptoms that can be due to medical nutrition and hydration. It is ethically permissible to discontinue medical nutrition and hydration that is contributing to suffering and prolonging the dying process [47]. Parents interviewed about their decision to forgo artificial nutrition and hydration (FANH) did not regret their decision [48]. This represented the paradoxical experience of not wanting their child to die yet concluding that FANH was the best of all options available, even viewed “as the only thing that made any sense”. The decision to FANH included that all children were viewed to have a significant alteration in quality of life due to pain and suffering and a decline in health that was not viewed as likely to improve. Family members also wrote about their experience regarding the harm they perceived when medical nutrition and hydration was used for their child with a neurodegenerative condition [49]. At such times, an approach of feeding to an amount that allows comfort can be beneficial and allow time to reflect on information while ensuring the child’s comfort. This topic is reviewed in greater detail elsewhere [47,48,50].

## 9. Conclusions

Vomiting, GI tract pain, and feeding intolerance are common problems in children with SNI. Awareness of sources with tests, sources due to the altered nervous system and risk for over-estimating calorie needs can then guide management strategies and lessen symptom burden in many. Some will progress to intractable feeding intolerance, likely due to further alterations in the CNS. Using the information in this review can improve comfort throughout life and lessen suffering at the end of life for children with SNI.

## Figures and Tables

**Table 1:** Chronic sources of retching, emesis, and visceral pain.

Cause	Management Options	Comments
Constipation	Polyethylene glycol Lactulose Milk of Magnesia Senna	Colonic distention from constipation can trigger pain symptoms due to visceral hyperalgesia and central neuropathic pain
GERD, motility disorders	H-2 blockers and PPIs Protective barrier: sucralfate Promotility drugs: erythromycin, metoclopramide Jejunostomy feeding tube	Motility disorders can be a result of impaired input from the CNS to the enteric nervous system Suggested by bloating, distension, retching, vomiting, discomfort Other problems can contribute, including constipation and pain
Vomiting reflex	Medications that block the 5HT-2, 5HT-3, H-1, Ach, and D-2 receptors Cyproheptadine (5HT-2, H-1, Ach) Ondansetron (5HT-3)	Suggested by retching, forceful vomiting, and associated symptoms of sweating, pale skin, and appearing distressed
Visceral hyperalgesia, central neuropathic pain	Gabapentin Pregabalin Tricyclic antidepressants	Suggested by pain, retching, and emesis associated with feedings, intestinal gas, flatus, and bowel movements
Autonomic dysfunction	Gabapentin Pregabalin Clonidine	Suggested by pain and emesis associated with tachycardia, hyperthermia, diaphoresis, and skin flushing
Pseudo-obstruction	Conservative management Erythromycin Neostigmine or pyridostigmine	Suggested by recurrent episodes of abdominal distension, pain, emesis, and severe constipation, in the absence of mechanical obstruction

Ach: acetylcholine; CNS: central nervous system; D: dopamine; H: histamine; 5HT: serotonin; GERD: gastroesophageal reflux disease; PPI: proton pump inhibitor.

**Table 2:** Interventions for chronic retching, vomiting, and visceral pain.

Intervention	Comments
Treat constipation	Minimizes colonic distension and further slowing of motility
Assess for over-feeding	Children at highest risk: intermittent hypothermia, minimal movement of extremities, decreased movement following symptom reduction, gradual health decline Initiate 30% reduction and monitor for 2–4 weeks
Review bolus volume and feed rate	Suggested guidelines: bolus < 15 mL/kg per bolus, continuous rate < 8 mL/kg/h [ 34]
Review osmolarity of feeds	Minimize use of elemental formulas or dilute, use additives to add calories without adding osmotic load (microlipid)
Gastric acid reduction and protective barrier: H-2 blockers, PPIs, sucralfate, antacids	Consider 8–12 weeks treatment course: chronic use of PPIs associated with *Clostridium difficile*, small bowel bacterial overgrowth, pneumonia, bone fracture, and hypomagnesemia Anticipate gastric acid rebound when a PPI is stopped; consider managing with short-term use of antacids or H-2 blocker
Gabapentin, pregabalin	Treatment of visceral hyperalgesia and dysautonomia
Tricyclic antidepressant	Treatment of visceral hyperalgesia and central neuropathic pain
Clonidine	Treatment of symptoms due to dysautonomia
Cyproheptadine	Blocks receptors that trigger the VC (5HT-2, H-1, and Ach)
Ondansetron	Blocks receptors that trigger the emetic reflex (5HT-3)
Erythromycin Metoclopramide	May improve gastric emptying and intestinal motility No clear benefit of one over the other Limit use of metoclopramide due to risk of dystonic reaction and lower seizure threshold
G-tube venting and equipment that allows venting during feedings	Minimizes gastric distension and associated discomfort
Gastrojejunal tube (GJ-tube)	Lessens gastric distension G-tube venting possible while feeding through the J-tube GI pain may not improve with J-tube feedings
Soy, partially hydrolyzed, or elemental formula	Management of protein hypersensitivity Higher omolarity with elemental formula
Select antibiotics	For *Helicobacter pylori* identified by stool antigen or endoscopy, or empirical trial for small bowel bacterial overgrowth
Anti-reflux surgery (fundoplication)	Consider empirical medication trials for problems outlined above before elective surgery Some children will develop retching, bloating, and pain following fundoplication

G-tube: gastrostomy tube; GJ-tube: gastrojejunal tube; TCA: tricyclic antidepressant; VC: vomiting center.
